# Urgent Alert: Potential Risk of Dengue Infection Transmission Through Blood Transfusion in Iran

**DOI:** 10.34172/aim.31756

**Published:** 2024-12-01

**Authors:** Zahra Taghinejad, Mohammad Asgharzadeh, Ali Akbar Pourfathollah

**Affiliations:** ^1^Department of Hematology and Blood Banking, Faculty of Basic Medical Sciences, Tarbiat Modares University, Tehran, Iran; ^2^Biotechnology Research Center and Faculty of Paramedicine, Tabriz University of Medical Sciences, Tabriz, Iran; ^3^Department of Immunology, Faculty of Basic Medical Sciences, Tarbiat Modares University, Tehran, Iran

**Keywords:** Dengue virus, Transfusion-transmissible dengue, Dengue viral infections

## Abstract

Dengue infection is an emerging public health issue in Iran, with about 149 confirmed newly infected cases. It can be transmitted by the bite of infected Aedes mosquitoes and even nosocomial routes. Due to the rapid replication and geographical spread of the mosquito, there is a potential risk of increased infected individuals. Given the possibility of the transmission of dengue infection through transfusion, it is important to implement policies to improve blood safety. Proper donor selection by utilizing appropriate blood donor questionnaires and performing general physical examinations, along with performing sensitive diagnostic tests on blood donor samples, utilizing pathogen reduction techniques, and implementing lookback programs, can be effective in reducing the risk of transfusion-transmitted dengue virus (TT-DENV).

## Introduction

 The dengue virus (DENV) is a small positive-stranded enveloped RNA arthropod-borne virus in the *Flaviviridae* family and the *Flavivirus *genus.^1^ It has four antigenically distinct serotypes with 47 strains, of which cause dengue fever in humans, and is mostly transmitted through the bite of infected female Aedes mosquitoes.^1^ Published evidence shows that DENV can also transmit through blood transfusions, needlestick injuries, organ or hematopoietic stem cell transplants, sexual contact, vertical route, and breast feeding.^2^

 Annually, there are 100 million cases of dengue infection across the world.^3^ DENV infection is also a now-a-days hot health issue in Iran.^4^ It can affect all age groups^3^ and is endemic in many developing and even developed countries.^5^ Clinical features of dengue infection may vary from an asymptomatic to a severe form.^3^ The incubation period for dengue infection is around 3 to 14 days^6^ and between 50% and 80% of cases of dengue infection are asymptomatic.^5^ Common clinical features in symptomatic cases are fever, headache, nausea, vomiting, myalgia, and flu-like symptoms.^7^

 The first case of transfusion-transmitted DENGV (TT-DENV) was reported in 2002 ^5^ and since then several case studies have reported TT-DENV.^8,9^ Despite stringent protocols to mitigate transfusion-transmitted infections, the diversity of pathogens, resource limitations, cost implications, and regulatory challenges make it impossible to carry out routine diagnostic tests for every infectious agent that can be spread through blood transfusions.^10,11^ Blood donation screening has not been routinely used for DENV in many regions,^12^ but several studies in endemic areas have found that more than 1 per 500 donations were DENV-RNA-positive.^8^

 Although transfusion-transmitted dengue has now been officially recognized,^13^ the increasing prevalence of DENV infection in Iran^4^ and the world^3^ necessitates immediate attention to the mechanisms of transmission, incidence rates, and effective preventive strategies. This review article aims to highlight the current research on epidemiology, transmission, pathophysiology, diagnosis, and management of DENV infection and provide actionable recommendations to mitigate the transfusion-transmitted DENV and enhance public health responses.

## Epidemiology of DENV Infection: A Global Overview with a Focus on Iran

 DENV represents the fastest-growing mosquito-borne viral disease in the world.^14^ More than 3.6 billion people in over 100 countries are at risk of DENV infection.^1^ The incidence of DENV infection has risen 30-fold over the past 50 years.^1^ The first recorded DENV outbreak was in 1779 in Jakarta, Indonesia, and Cairo, Egypt.^1^ Each year, dengue epidemics happen in the Americas, Africa, Asia, and Australia, with significant outbreaks in Southeast Asia following World War II, driven largely by urbanization.^1,14^ Notably, the largest DENV outbreak in the United States occurred in 2016, with over 2.38 million reported cases.^1^

 In Iran, the presence of DENV is primarily due to the geographical proximity to endemic regions such as Afghanistan and Pakistan, along with the spread of the Aedes mosquito, the primary vector.^15^ This viral disease has become an important health concern in Iran since 2008, when the first case was confirmed in a 61-year-old man with the history of traveling to Malaysia.^15^ Since that time, sporadic cases of DENV have been reported, particularly in the Sistan and Baluchestan province and southern regions near the Persian Gulf.^15^ As of 2024, the first detected case dated back to May 26, from a traveler who had returned from the United Arab Emirates.^16^ Until July 9, 2024, dengue fever was detected in four provinces of Iran, with 149 confirmed cases.^16^ The detection of the Aedes mosquitoes in different towns across Iran and the increasing number of infected cases have created public concern and highlighted the need for increased surveillance and preventive measures.^17^

## Dengue Disease

**Figure 1:**
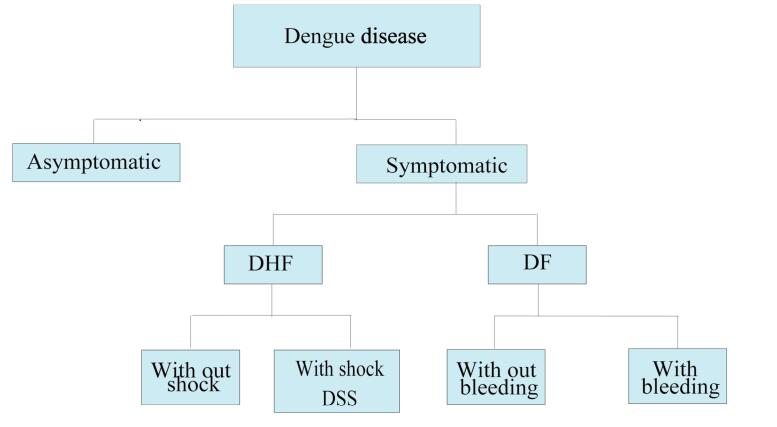
Dengue Disease. Classification of Dengue Disease based on Severity. DF: dengue fever, DHF: dengue hemorrhagic fever, DSS: dengue shock syndrome

 Dengue disease can be sub-classified into several distinct clinical forms based on severity^14^ (Figure 1). Dengue fever (DF) is an acute and self-limiting form of symptomatic DENV infection.^14^ DF may occur with or without hemorrhagic symptoms.^14^ It is distinguished by high fever, headache, retro-orbital pain, rash, and musculoskeletal pain.^18^ Some patients may also show mild hemorrhagic symptoms, including petechiae, mucosal bleeding, or a positive tourniquet test.^18^ In more severe forms, dengue hemorrhagic fever (DHF) occurs. It can be further classified into two categories: cases with shock, termed dengue shock syndrome (DSS), and cases without shock.^19^ DHF is characterized by increased vascular permeability, thrombocytopenia, and various hemorrhagic signs.^20^ These issues may lead to complications such as bleeding, plasma leakage into peritoneal spaces, and, in severe cases, hypovolemic shock.^18^ Different risk factors like secondary infections, severe viremia, pre-existing comorbidities, and pediatric age groups have been related to the development of the DHF/DSS.^19^

## DENV Transmission

 Viruses mainly spread from one host to another due to the nature of the reaction of a virus and the host and the number of infected people.^21^ DENV transmission is a compound process influenced by different factors such as mosquito vectors, climate conditions, travel, immune system status, donor selection, and public awareness.^22^ The DENV is mainly transmitted through *Aedes aegypti* or *Aedes albopictus* mosquito vectors.^23^ These mosquito vectors can be identified by black and white markings on the legs.^19^ It is estimated that the mosquito can lay eggs about three times in its lifetime while producing about 100 eggs at a time.^24^ These eggs can survive dry periods for a number of months and can receive DENV from the previous generation.^24^ Recent research has shown that immediate mechanical transmission by *Aedes aegypti* mosquitoes may take place without the requirement for viral replication in the mosquito, thus producing faster and bigger outbreaks.^23^

 Apart from these mosquito-borne cases, DENV has been described to be transmitted through blood transfusions, needlestick injuries, organ or hematopoietic stem cell transplants, sexual contact, vertical route, and breast feeding.^2,25^ The first case of TT-DENV was reported in 2002 in a 76-year-old woman with megaloblastic anemia who experienced a mild fever two days after a blood transfusion.^5^ In this case, the donor was a 17-year-old boy with a skin rash seven days after blood donation. DENV was detected in the donor blood, and the post-transfusion blood sample of the recipient was serologically positive for dengue-specific immunoglobulin M (IgM).^5,26^

 The transmission of DENV through red blood cells and fresh frozen plasma (FFP) was reported in Singapore.^27^ In this report, the recipient of the packed cell developed a fever on day 2 post-transfusion, and the other recipient of the FFP developed a high fever with a pleural effusion on day 1 post-transfusion.^5^ The transmission of DENV through platelet (PLT) concentrates was reported in a case study in Brazil in a 56-year-old man with aplastic anemia. He received one PLT pheresis unit from a regular platelet donor, and five days after transfusion, he had daily high fever (39.5 °C) and arterial hypotension.^28^

 Transmission of DENV through needlestick injuries has been reported in several studies.^2,29^ Although rare, there have been few cases of DENV transmission through hematopoietic stem cell transplantation.^30^ It can also be transmitted through solid organ transplant; however, there have been no reported cases of graft rejection.^31^

## DENV Life Cycle

 The life cycle of the DENV involves several intricate stages within the mosquito vector and the human host.^21^ In mosquitoes, DENV initially infects the midgut by binding to receptors such as heat shock protein (Hsp)-70.^32^ After replication in the midgut, the virus disseminates to various body compartments, like salivary glands.^32^

 In humans, the life cycle of a virus begins with the entrance of the virus into the bloodstream.^33^ The virus primarily targets mononuclear phagocytic cells, including monocytes, macrophages, and dendritic cells, as well as skin-resident Langerhans cells. DENV utilizes molecules like Heparan sulfate, phosphatidylserine families, and Hsp-90 to enter the host cells through Clathrin-mediated endocytosis.^33^ Inside the cell, the viral nucleocapsid is uncoated, and the RNA genome is replicated and translated into a single polyprotein. New viral particles are then assembled and released into the bloodstream through exocytosis.^33^ These particles can spread to various organs and tissues, resulting in the clinical manifestations of DF or DHF/DSS.^33^ The cycle is completed when another female Aedes mosquito bites an infected human. After virus ingestion and replication in the mosquito, the virus can be transmitted to other human hosts through mosquito bites.

## Pathophysiology of DENV Infection

 The host immune response against DENV determines the severity and pathophysiology of the infection.^34^ In innate immune response, pattern recognition receptors (PRRs) and cytoplasmic retinoic acid-inducible gene I (RIG-I) detect viral RNA. This immune detection sets off a cascade of events, including mitochondrial antiviral signaling and the production of type I interferons (IFNs) and pro-inflammatory cytokines.^34^ These cytokines recruit various immune cells, including natural killer cells and T cells, to eliminate the infection.^35^

 The cell-mediated arm of the adaptive immune system consists of a cluster of differentiation (CD)-4^+^ helper T cells and CD8^+^ cytotoxic T cells.^36^ They are particularly crucial for coordinating the immune response through promoting cytokine release, boosting macrophage and B cell activation, and killing infected cells. On the other hand, B lymphocytes, differentiate into plasma cells that produce antibodies targeting DENV after their activation by viral antigens and CD4^+^T Cells.^35^ The primary humoral adaptive immune response focuses heavily on viral envelope protein, which is essential for the virus to enter host cells.^34^

 A secondary dengue infection, particularly with a different serotype, complicates matters through a process known as antibody-dependent enhancement (ADE).^34^ In this scenario, pre-existing antibodies from the primary infection may not effectively neutralize the new serotype.^35^ Instead, they can bind to the new virus and facilitate its entry into immune cells like monocytes and macrophages.^34^ This enhanced viral entry can increase viral replication within these cells.^35^ Higher viral load and overactive immune response increase the risk of DHF and DSS through cytokine storm, and disrupted endothelial function.^34^

 To stabilize the infection, DENV has been shown to interfere with innate and adaptive immune signaling via different mechanisms like inhibition of type I IFN production and signaling, inhibition of antigen presentation, inducing antigenic variation, and ADE.^35^

## Clinical Manifestations of DENV Infection and its Diagnosis

 Clinical manifestations vary in people infected with DENV due to their immune and physiological status.^19^ After an incubation time of 3-7 days, commonly observed symptoms in DENV infection include fever, headache, myalgia, vomiting, nausea, thrombocytopenia, raised liver transaminases, and leukopenia.^7^ The clinical course of DENV infection is divided into three febrile, critical, and recovery phases.^14^ Febrile phase is 3-7 days with a high body temperature, myalgia, backache, headache, upper respiratory tract symptoms, leukopenia, thrombocytopenia, and raised liver transaminases.^37^ The critical phase is seen in a proportion of patients; in other words, it is only seen in patients with DHF/DSS.^14^ This course is characterized by systemic vascular leakage indicated by an increase in hematocrit levels and hypoalbuminemia.^20^ It can be a life-threatening stage of DENV infection because of the increased risk of bleeding, myocarditis, retinitis, encephalitis, nephritis, and hemorrhagic liver necrosis.^6,38,39^ In the recovery phase, patients experience marked improvement in general health status and bradycardia, named recovery bradycardia.^14^ Due to the severe impact of DENV infection on the body, timely diagnosis, monitoring, and appropriate prevention principles are essential in preventing serious complications.^39^

 Diagnostic techniques for DENV include viral nucleic acid, antigens, or antibodies used singly or in combinations. Some common tests used in the diagnosis of DENV are enzyme-linked immunosorbent assays (ELISA), reverse transcriptase polymerase chain reaction (RT-PCR) assay, nucleic acid amplification tests (NAATs), immuno-chromatographic tests, tourniquet test and rapid low-resource serotype-specific tests.^40^ In the early febrile phase of DENV infection, detection of viral antigens or nucleic acids in the plasma is highly sensitive.^14^ Testing for anti-DENV IgM and/or IgG antibodies by ELISA is the most frequently applied method for confirmation of DENV infection.^41^ In primary infections, anti-DENV IgM and IgG can be detected from 5 and from 10–15 days after the onset of illness, respectively.^42^ In secondary infections, IgM appears earlier with lower titers than in primary infection, and the remaining titer of IgG from the previous infection increases rapidly.^42^ Since the production of antibodies takes time, their use alone would miss asymptomatic patients in the early course of illness. Thus, it is essential to utilize a combination of methods to detect DENV infections, especially in donors.^5^

## Preventive Measures for Reducing the Risk of DENV Infection, Especially TT-DENV

 As the best way to prevent the disease is to block the transmission routes of DENV, vector control, increasing public information on viral transmission routes, and utilizing mosquito repellents and adequate clothing are major preventive strategies.^19,43^

**Figure 2:**
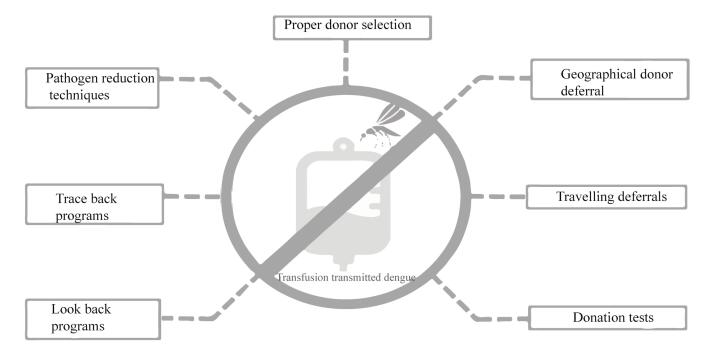
Ways to Reduce the Transfusion Transmitted Dengue Virus (TT-DENV) in Iran

 To reduce the frequency of TT-DENV, several strategies can be implemented based on the research findings. Due to the limitations of the screening methods, a combination of geographical donor deferrals, traveling deferrals, donation testing, pathogen reduction techniques, and lookback programs can be utilized (Figure 2).^44^ Blood donor selection is the first step in maintaining blood safety. It allows for considerable reduction of risks through deferrals for donation.^45^ Deferral criteria may be applied to donors if they have traveled to DENV-endemic regions, experienced undiagnosed febrile illness, have scars of mosquito bites, or have had a history of DENV infection within 120 days of symptom resolution.^46^ Asymptomatic patients with a history of travel to DENV endemic regions should be deferred for 28 days upon returning to non-endemic places.^46^ Donation testing through NAT or screening for viral antigens or IgM antibodies can be considered to increase blood safety.^5^ For plasma and PLT pheresis donations, the amotosalen/UVA pathogen reduction system may also be a good consideration.^47^

 In order to prevent further transmission of DENV through other products of a DENV-infected donor, clinicians should suspect dengue in cases of transfusion-associated fever and implement traceback strategies.^5^ Utilizing lookback strategies can also be effective in improving blood safety^48^ and preventing TT-DENV.

 Regular donors should also protect themselves from mosquito bites by wearing protective clothing or using insect repellents.^43^ They also should increase their awareness about the transmission routes of DENV infection.

## Management of DENV

 Currently, there is no approved antiviral drug specifically for DENV, and each country has established its own management protocols.^14^ It is recommended that patients maintain adequate oral fluid intake and take paracetamol in the febrile phase.^49^ In the critical phase, effective management of DF relies on fluid resuscitation, ensuring that the rate of fluid administration corresponds to the plasma leakage.^49^ Platelet transfusions are necessary for patients with really severe hemorrhagic symptoms with thrombocytopenia or patients needing emergency surgery.^14^

**Figure 3:**
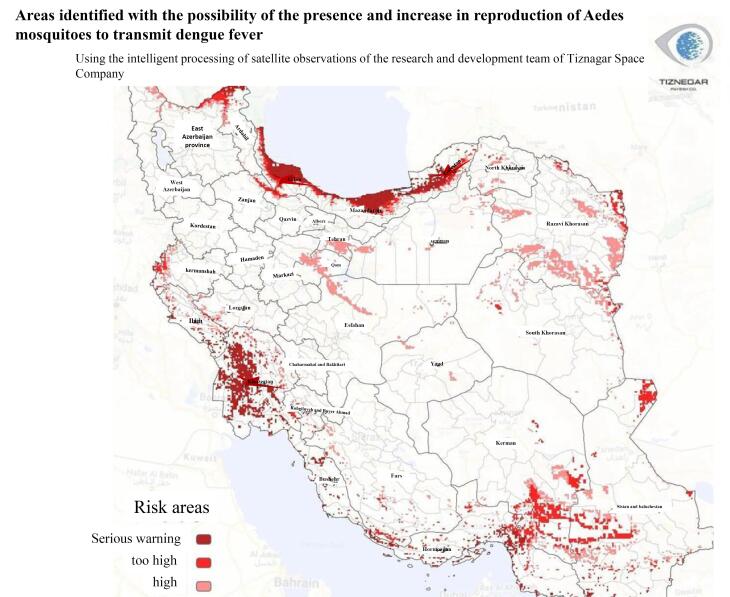
Possible Distribution Map of Aedes Mosquito and DENV Infection in Iran. The picture was uploaded by the research and development team of Tiznegar Institute.^50^ DENV: dengue virus

 Due to the rapidly growing threat of increased DENV infection rates in Iran because of the rapid replication and geographical spread of the mosquito (Figure 3), the public and healthcare workers should be better informed about DENV and TT-DENV infection.

## Conclusion

 Transmission of DENV infection through the transfusion of blood and blood components has previously been reported in studies. Due to the importance of improving blood safety, sufficient attention from the ministry of health to prevent TT-DENV is vital at this point in time. Controlling TT-DENV is possible with proper donor selection by performing appropriate deferrals, rapid diagnosis, utilizing pathogen reduction techniques, and implementing lookback programs. Controlling the mechanical spread of disease through increasing public awareness and eliminating vectors can also reduce TT-DENV indirectly.
